# Studies on the Control of Ascochyta Blight in Field Peas (*Pisum sativum* L.) Caused by *Ascochyta pinodes* in Zhejiang Province, China

**DOI:** 10.3389/fmicb.2016.00481

**Published:** 2016-04-12

**Authors:** Na Liu, Shengchun Xu, Xiefeng Yao, Guwen Zhang, Weihua Mao, Qizan Hu, Zhijuan Feng, Yaming Gong

**Affiliations:** ^1^Institute of Vegetables, Zhejiang Academy of Agricultural SciencesHangzhou, China; ^2^Institute of Vegetable Crops, Jiangsu Academy of Agricultural Sciences/Jiangsu Key Laboratory for Horticultural Crop Genetic ImprovementNanjing, China; ^3^Center of Analysis and Measurement, Zhejiang UniversityHangzhou, China

**Keywords:** field pea, ascochyta blight, *Ascochyta pinodes*, fungicides, biological control

## Abstract

Ascochyta blight, an infection caused by a complex of *Ascochyta pinodes, Ascochyta pinodella, Ascochyta pisi*, and/or *Phoma koolunga*, is a destructive disease in many field peas (*Pisum sativum* L.)-growing regions, and it causes significant losses in grain yield. To understand the composition of fungi associated with this disease in Zhejiang Province, China, a total of 65 single-pycnidiospore fungal isolates were obtained from diseased pea samples collected from 5 locations in this region. These isolates were identified as *Ascochyta pinodes* by molecular techniques and their morphological and physiological characteristics. The mycelia of ZJ-1 could penetrate pea leaves across the stomas, and formed specific penetration structures and directly pierced leaves. The resistance level of 23 available pea cultivars was tested against their representative isolate *A. pinodes* ZJ-1 using the excised leaf-assay technique. The ZJ-1 mycelia could penetrate the leaves of all tested cultivars, and they developed typical symptoms, which suggested that all tested cultivars were susceptible to the fungus. Chemical fungicides and biological control agents were screened for management of this disease, and their efficacies were further determined. Most of the tested fungicides (11 out of 14) showed high activity toward ZJ-1 with EC_50_ < 5 μg/mL. Moreover, fungicides, including tebuconazole, boscalid, iprodione, carbendazim, and fludioxonil, displayed more than 80% disease control efficacy under the recorded conditions. Three biocontrol strains of *Bacillus* sp. and one of *Pantoea agglomerans* were isolated from pea-related niches and significantly reduced the severity of disease under greenhouse and field conditions. To our knowledge, this is the first study on ascochyta blight in field peas, and results presented here will be useful for controlling the disease in this area.

## Introduction

Ascochyta blight (more commonly known as “black spot disease”) is one of the most severe diseases of field peas, and it is distributed worldwide, including almost all of the major pea-growing areas (Bretag et al., 2006). Yield losses caused by ascochyta blight in peas have been estimated to be at least 10% and up to 60% each year in Australia (Bretag et al., 2006), 40% in experimental field plots in France (Tivoli et al., 1996) and up to 50% in field trials in Canada (Wallen, 1965, 1974; Xue et al., 1996). China has remained the leading green pea-producing country over the last decade, and China produced more than 60% of the total green peas in the world in 2013 (Figure S1A). In addition, China was the second-largest dry pea-producing nation after Canada, and dry pea production reached 1566 kilotons in 2013 (Figure S1B) (FAO, 2015)^1^. However, few studies have been conducted to characterize the fungi involved in ascochyta blight in peas and disease management in China. Considering that peas are an economically important crop in China, the economic losses caused by ascochyta blight are significant and demand more attention.

Ascochyta blight is caused by a complex of fungal pathogens, commonly referred to the ascochyta complex, including *Ascochyta pinodes* L.K. Jones (teleomorph: *Mycosphaerella pinodes* (Berk. & Blox.) Vestergr.), *Phoma medicaginis* var. *pinodella* (L.K. Jones) Morgan-Jones & K.B. Burch, *Ascochyta pisi* Lib. (teleomorph: *Didymella pisi* sp. nov.) and *Phoma koolunga* Davidson et al. sp. nov. (Davidson et al., 2009; Liu et al., 2013). This blight complex causes a range of different symptoms, including ascochyta blight, foot rot, black stem and leaf and pod spot. Seed quality may also be reduced through seed discoloration or retardation of seed development. *A. pinodes* can infect seedlings and all aerial parts of pea plants, causing necrotic leaf spots, stem lesions, shrinkage and dark-brown discoloration of seeds, blackening of the base of the stem, and foot rot in seedlings. The disease symptoms caused by *P*. *pinodella* are similar to those observed with *A. pinodes*. However, *P*. *pinodella* infection can result in more severe foot rot symptoms that can extend below ground, while causing less damage to the leaves, stems and pods. *A. pisi* causes slightly sunken, circular, tan-colored lesions with dark brown margins that occur on the leaves, pods, and stems (Chilvers et al., 2009). This fungus usually does not attack the base of pea plants or cause foot rot. *P. koolunga* presents disease symptoms on pea seedlings that are indistinguishable from those caused by *M. pinodes*, other than a 24 h delay in disease development under controlled conditions (Davidson et al., 2009). Recently, *Phoma herbarum* and *Phoma glomerata* have also been shown to be associated with the ascochyta blight complex on field peas in Australia, causing typical dark brown lesions and chlorotic halos on pea leaves (Li et al., 2011; Tran et al., 2014). All related pathogens are seed-borne pathogens that can also survive on infected pea debris.

Using resistant cultivars for the management of ascochyta blight in peas would be the most practical, effective and economical approach. Unfortunately, sources of resistance to the ascochyta blight fungi are very limited, and cultivars that are highly resistant to ascochyta blight have not yet been developed. Although some potential resistance sources have been found in Canada (Xue and Warkentin, 2001), New Zealand (Kraft et al., 1998), and the United Kingdom (Clulow et al., 1991), these pea lines were found to have moderate resistance and did not tolerate all fungi species of the ascochyta complex. Control of ascochyta blight is largely dependent on fungicide treatment and cultural practices such as crop rotation. Fungicides, including mancozeb, chlorothalonil, benomyl, carbendazim, and thiabendazole, have been used to effectively control ascochyta blight and increase yield (Warkentin et al., 1996, 2000; Bretag et al., 2006). However, the baseline sensitivity of the fungi associated with ascochyta blight has been shown to be isolate specific. Fungicide dosages need to be optimized for field control in different areas.

Fungicide applications, however, may increase production costs, reduce yield quantities due to the residues, and also pose a risk to the environment due to drift into non-target areas. Moreover, the intensive application of fungicides can lead to the emergence of fungal strains that are resistant to commercial chemicals. It has been reported that the some *Ascochyta rabiei* isolates, pathogens of chickpea ascochyta blight, exhibited a mean 100-fold increase in resistance to the QoI (strobilurin) fungicides azoxystrobin and pyraclostrobin when compared to sensitive isolates (Chang et al., 2007; Wise et al., 2008). The above limitations have prompted us to explore safer and more environmentally friendly biological control measures for ascochyta blight in field peas as alternatives. Bacterial antagonists *Pseudomonas fluorescens, Bacillus* spp. and *Serratia* spp. significantly reduced the severity of ascochyta blight in peas under greenhouse conditions (Wang et al., 2003). The mycoparasite *Clonostachys rosea* strain ACM941 was an effective bioagent in controlling pea root rot complex caused by *A. pinodes, Rhizoctonia solani* and other six pathogenic fungi (Xue, 2003).

Ascochyta blight in field peas occurs and has become more common in fields in Zhejiang Province, a main pea-producing area in China, during the last decade. It has caused approximately 10–30% peas yield losses in this area every year. However, little has been known about the pathogen(s) involved in ascochyta blight and management of this disease in this area until now. The objectives of this current study were to (i) identify and characterize ascochyta blight pathogens in this area; (ii) evaluate the susceptibility of 23 pea cultivars to ascochyta disease; (iii) screen commercial fungicides for disease management; and (iv) investigate the use of antagonistic bacterial agents for disease control.

## Materials and methods

### Ascochyta blight-associated fungal isolates

Field pea plant tissues with typical ascochyta blight symptoms were collected from fields in Taizhou, Lanxi, Quzhou, and Lishui as well as our pea-breeding trial sites in Haining. All sites are located in Zhejiang Province (29°12′N, 120°30′E) in China. Infected leaves and stems were cut into pieces and surface sterilized with a 30 s treatment in 70% ethanol followed by 15 min in sodium hypochlorite (10% active chlorine) and three subsequent washing steps with sterile water for at least 15 min each. Sterilized samples were placed onto potato dextrose agar (PDA) plates (200 g potato, 20 g glucose, 15 g agar, and 1 L water) supplemented with chloramphenicol (10 μg/ml) and kanamycin (25 μg/ml) and incubated at 25°C for 3 days. After incubation, the edges of fungal colonies were cut out and transferred to new plates for purification. Single conidium-derived isolates were prepared and saved at 4°C for further study.

### Fungal morphological and physiological characters

The morphological and physiological features of ZJ-1 isolate were compared on five nutrient media, including complete medium (CM) (10 g glucose, 2 g peptone, 1 g yeast extract, 1 g casamino acids, nitrate salts, trace elements, 0.01% of vitamins, 15 g agar and 1 L water, pH 6.5); Warkingsman agar (WA) (5 g peptone, 10 g glucose, 3 g meat extract, 5 g NaCl, 15 g agar, and 1 L water, pH 6.5) (Berg et al., 2005); Pea leaves agar (PLA) (200 g pea leaves, 20 g glucose, 15 g agar, and 1 L water); Oatmeal agar (OA) (20 g oatmeal, 15 g agar and 1 L water) and PDA. Linear growth rate of ZJ-1 on above media at 25°C was determinate in three replicate plates (9 cm) by measuring the two diameter of the colony every day over a 9-day period. To visualize the surface differentiation of colonies on various media, a droplet of 2.5% bromophenol blue water solution was placed on the colony surface. The production of pycnidia was studied on the 1/3 PDA agar plates. After 10 days incubation at 25°C under 16 h light and 8 h dark condition, the character of spore was visualized by Leica TCS SP5 imaging system (Wetzlar, Hesse-Darmstadt, Germany). For better views of septum, the spore was stained with calcofluor white.

### DNA isolation and molecular identification

For DNA extraction, isolates were grown in potato dextrose broth for 1 day. Mycelia were then harvested and washed with sterilized water. Genomic DNA was extracted using a previously published protocol (Saitoh et al., 2006). The primers ITS1F (5′-TCCGTAGGTGAA CCTGCGG-3′) and ITS4R (5′-TCCTCCGCT TATTGATATGC-3′) were used to amplify the ITS (internal transcribed spacer) region (White et al., 1990). Primers AscGAPDH-F (5′-GCAACGCGTGAG TAACTCTCA-3′) and AscGAPDH-R (5′-TGTTGA CACCCATAACGAACA-3′) were designed in this study to amplify a 496 bp PCR product from the *G3PDH* (glyceraldehyde 3-phosphate dehydrogenase) gene from *Ascochyta* species. PCR amplifications were performed in a 50 μl reaction volume using the Platinum® Pfx DNA Polymerase (Thermo Fisher Scientific Inc.). The PCR products were gel purified using a QIAquick gel extraction kit (Qiagen) and sequenced at BGI (Shanghai, China).

### Scanning electron microscope for infection patterns

The infected leaves were cut into small pieces and first fixed with 2.5% glutaraldehyde in phosphate buffer (PBS) (0.1 M, pH7.0) overnight. Then, samples were washed three times and post-fixed with 1% OsO4 in PBS for 2 h. After double fixation, the samples were dehydrated in a graded series of ethanol (30, 50, 70, 80, 90, and 100%) for 20 min at each step and then transferred to pure isoamyl acetate (v:v = 1:1) overnight. Finally, the samples were dehydrated in a Hitachi Model HCP-2 critical point dryer with liquid CO_2_. The dehydrated samples were coated with gold-palladium in a Hitachi Model E-1010 ion sputter for 5 min and observed in a Hitachi Model TM-1000 SEM.

### Pea cultivar susceptibility

The excised leaf assay was used to assess pea cultivar susceptibility to the ZJ-1 strain (Wang et al., 2000). Twenty-three cultivars that we collected from natural populations in Zhejiang, Gansu, Sichuan, Anhui, and Hubei provinces of China were evaluated for their susceptibility to the ZJ-1 strain (Table 1). Their origin and cultivars characters were described in Table 1. Five millimeter (diameter) mycelial plugs were taken from the edge of a 7-day old colony grown on PDA and were transferred onto pea leaves for testing. After inoculation, the leaves were moved into 9 cm petri dishes covered with sterilized wet tissue for moisture. Eight excised leaves in four Petri dishes were used to assess cultivar susceptibility. The assay was repeated three times. Lesion radii were measured after a 3-day incubation at 25°C. The data were analyzed using Fisher's protected least significant difference test (*P* = 0.05) in SAS (SAS version 8.0; SAS Institute, Cary, NC, USA).

**Table 1:** **Origin and morphological characters of 23 pea cultivars and their susceptibility to ***Ascochyta pinodes*** ZJ-1 using the excised leaf assay in laboratory tests**.

**Cultivar**	**Origin**	**Plant height**	**Cotyledon color**	**lesion radii (cm)**
D8341	Gansu	Tall	Green	4.08±0.30 a
CH-KSKT	Gansu	Tall	Yellow	3.88±0.25 a
Zhejiang-3	Zhejiang	Dwarf	Green	3.75±0.29 a
GS-28	Gansu	Tall	Yellow	3.75±0.50 a
Chaoxiang wan	Zhejiang	Dwarf	Green	3.58±0.43 a
GS-23	Gansu	Tall	Green	3.50±0.41 a
Anhui-1	Anhui	Dwarf	Yellow	3.50±0.41 a
GS-25	Gansu	Tall	Green	3.00±0.00 b
J-14	Sichuan	Tall	Yellow	2.88±0.48 bc
J-16	Sichuan	Tall	Green	2.63±0.48 bcd
GS-39	Gansu	Tall	Green	2.38±0.48 cde
Mizhu-9	Gansu	Dwarf	Green	2.38±0.48 cde
Tengfei-5	Gansu	Tall	Green	2.38±0.48 cde
JQ-3	Sichuan	Dwarf	Green	2.33±0.46 cde
Zhejiang-1	Zhejiang	Tall	Green	2.25±0.29 def
Cuizhu	Gansu	Tall	Green	2.13±0.25 def
Xiangwan-1	Zhejiang	Dwarf	Green	2.13±0.25 def
SUA-1	Hubei	Tall	Green	2.13±0.48 def
landzea	Hubei	Tall	Green	2.13±0.25 def
Zhewan-1	Zhejiang	Tall	Green	2.00±0.00 ef
JP-2	Sichuan	Tall	Yellow	2.00±0.00 ef
J-210	Sichuan	Tall	Green	1.90±0.27 ef
Zhengzhu Lv	Gansu	Tall	Green	1.70±0.24 f

*The data were analyzed using Fisher's protected least significant difference test (P = 0.05) in SAS (SAS version 8.0; SAS Institute, Cary, NC, USA). The same letters are not significantly different (P = 0.05)*.

### Determination of baseline EC_50_ values

To test the susceptibility of the ZJ strain to 14 commonly used commercial fungicides (eight fungicide categories, Table 2), we determined the EC_50_ of all tested fungicides against ZJ-1.A 5 mm mycelial plug from each strain was transferred onto a PDA agar plate containing a fungicide (tebuconazole, pyrimethanil, propiconazole, carbendazim, iprodione, boscalid, prothioconazole, penthiopyrad, chlorothalonil, thiophanate-methyl and prochloraz) at 0.1, 0.25, 0.5, 1, 2.5, 5, or 10 μg/mL. To determine the EC_50_ of fungicides, including tridemorph, fludioxonil and difenoconazole, serial concentrations of 0.005, 0.01, 0.02, 0.05, 0.075, 0.1, and 0.25 μg/mL were tested. The solvent dimethyl sulfoxide was used as a negative control treatment. Four (6 cm) replicated plates were used for each concentration. Plates were then placed in an incubation chamber at 25°C. When the ZJ-1 colony on the negative control plate extended to two-thirds of the plate, mycelial growth on each plate was recorded. EC_50_ values were calculated using the DPS (Data Processing System) computer program (Hangzhou Reifeng Information Technology Ltd., Hangzhou, China). To determine whether the EC_50_ of ZJ-1 was representative of the susceptibility of all *Ascochyta pinodes* isolates, five isolates were randomly picked from the remaining 64 isolates and tested for growth inhibition on PDA agar plates supplemented with individual fungicides at the EC_50_ concentration of the ZJ-1 strain. The experiment was repeated three times.

**Table 2:** **Toxicity of 14 fungicides against ***Ascochyta pinodes*** ZJ-1**.

**Fungicides categories**	**E-ISO**	**Regression equation**	**EC_50_ (μg/mL)**	***r***
Benzimidazole	Carbendazim	Y = 3.447X+4.587	1.318	0.999
Benzimidazole	Thiophanate-methyl	Y = 4.442X-0.369	16.163	0.907
Sterol Biosynthesis Inhibiting (SBIs)	Tridemorph	Y = 0.490X+6.140	0.005	0.976
SBIs	Difenoconazole	Y = 1.796X+6.412	0.167	0.989
SBIs	Prochloraz	Y = 1.515X+5.919	0.248	0.987
SBIs	Tebuconazole	Y = 1.424X+5.481	0.459	0.982
SBIs	Propiconazole	Y = 1.011X+4.939	1.15	0.996
SuccinateDehydrogenase Inhibitors (SDHI)	Boscalid	Y = 1.447X+4.504	2.201	0.994
SDHI	Penthiopyrad	Y = 0.714X+4.435	6.178	0.971
Phenylpyrrole	Fludioxonil	Y = 3.057X+8.763	0.058	0.992
Phyrimidine	Pyrimethanil	Y = 3.069X+5.153	0.891	0.972
Dicarboximides	Iprodione	Y = 1.719X+4.633	1.635	0.976
Triazolinthione	Prothioconazole	Y = 1.279X+4.103	5.026	0.995
Substitutive Benzene	Chlorothalonil	Y = 0.781X+4.106	13.969	0.987

### Isolation of antagonistic bacterial agents against ZJ-1

Bacterial isolates were recovered from the leaves, stem tissues, roots and rhizosphere soil of peas grown in the above-mentioned five fields using dilution plating methods (Barraquio et al., 1997). Briefly, each sample was homogenized with a sterilized mortar and pestle. Macerated samples were serially diluted with sterile 0.85% NaCl solution, and resulting suspensions were plated onto LB agar. Single colonies were randomly picked according to colony morphology from plates after 48 h incubation at 30°C and stored at −70°C for further investigation. The antifungal activity of pea-associated bacterial strains against ZJ-1was conducted on Waksman's Agar (WA) plates. All isolates were tested in triplicate. After plates were incubated at 25°C for growth, the inhibition zone of each bacterial isolate was examined until the colony of target fungal pathogen in the control extended to more than two-thirds of the plate. The non-antagonistic activity of *Bacillus subtilis* strain PY79 was used as a control strain. For bacterial identification, the 16S ribosomal DNA (rDNA) fragment of bacterial isolates was amplified using the primer pair fD1/rP2 (Weisburg et al., 1991). The PCR products were purified and sequenced by BGI Co. (Shenzhen, China).

### Efficacy of fungicides and biocontrol agents under greenhouse and field conditions

The “GS-28,” a field pea cultivar that is susceptible to ascochyta blight, was used to test the efficacy of fungicides and biocontrol agents under greenhouse and field conditions. Under greenhouse conditions, seeds were sown in pots (8 cm × 9 cm × 12 cm; two seeds per pot) filled with a complex fertilizer soil. The pots were kept in a glass greenhouse at 25°C (day) and 18°C (night). For each treatment, there were 3 replicates with 15 pots per replicate. After 4 weeks of growth, the seedlings were sprayed with a fungicide or a cell suspension of biocontrol agents with a hand-held atomizer until numerous droplets were deposited onto the surface of leaves. After droplets on the leaves air-dried for 12 h, each treated plant was sprayed until run-off with a spore suspension of ZJ-1, which was collected from 2-week-old 1/3 PDA plates and resuspended at a concentration of 10^5^ conidia per mL in 0.05% Tween 20. The treatment without fungicides or antagonist bacteria application but inoculated with the ZJ-1 spore suspension was used as a control. Disease severity on the plant leaves and stems was rated 2 weeks after inoculation. The test of the efficiency of the fungicides and biocontrol agent was repeated twice under greenhouse condition.

To test the efficiency of disease control in the field, seeds were sown into soil in November, 2014, and disease control agents were applied in March, 2015. The fields were located in Haining, where ascochyta blight was occurring and causing severe losses every year. The treatments, both fungicides and bacterial agents, were applied twice, at the initiation of flowering and mid-flowering during the growing season. The field trials were conducted using a randomized plot design with three replicates of each treatment. Each plot was 4 × 5 m^2^ in size. Appropriate fertilizers and herbicides were applied according to standard management practices. Disease severity on the plant leaves and stems was rated 2 weeks after the second application. A total of 30 pea seedlings were randomly chosen for disease severity survey in each plot.

Based on the efficacy of fungicides according the EC_50_ and their cost, five fungicides, including the tebuconazole, boscalid, iprodione, carbendazim, and fludioxonil, were tested in this study. The dosage of the tebuconazole, boscalid and iprodione fungicides was 125 g/ha, while the doses of carbendazim and fludioxonil were 900 and 40 g/ha, respectively. Fungicides were applied in a water volume of 700 L/ha. For the bacterial agents, *Bacillus* sp. strains and *Pantoea agglomerans* were grown in Landy et al. (1948) and King et al. (1954), respectively, on a shaker (200 rpm) at 30°C for 3 days. Each plot was sprayed twice with 1 L of bacterial cell suspension at concentration of 10^8^ CFU/mL with 0.05% Tween 20. Symptoms on foliage were visually estimated using a 0-to-5 scale (Zhang et al., 2003; Liu et al., 2013). The disease severity (Ds) for each plot was calculated using the formula [∑number of peas in each class × each evaluation class)/(total number of pea × 5)] × 100. The biological efficacy for each treatment was determined by applying Abbott's formula: [(Ds of the negative control − Ds of the treatment)/Ds of the negative control)] × 100%. The data were analyzed using Fisher's protected least significant difference test (*P* = 0.05) in SAS (SAS version 8.0; SAS Institute, Cary, NC, USA).

## Results

### Ascochyta blight pathogen(s) isolation and identification

**Figure 1:**
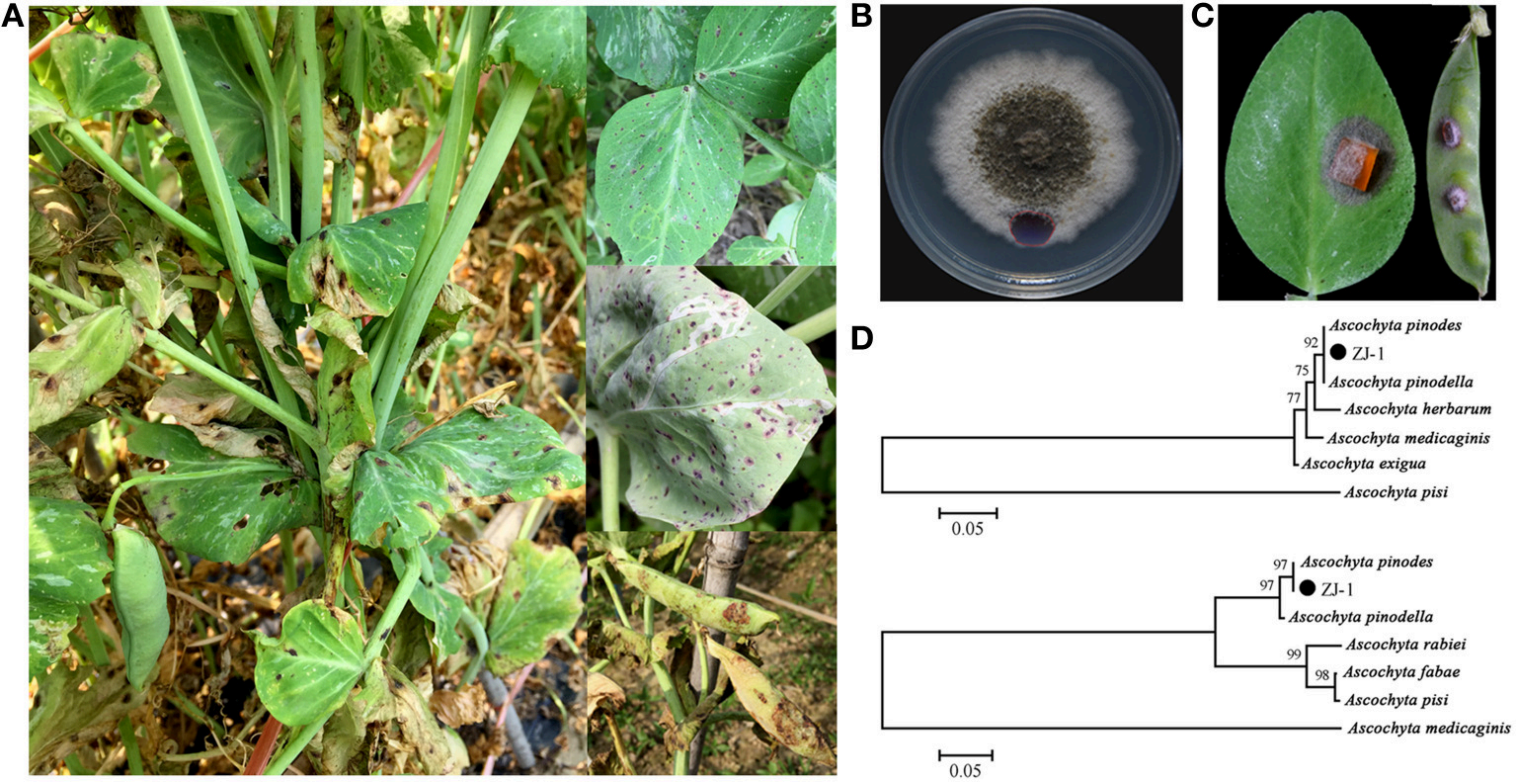
**Disease symptoms of ascochyta blight of field peas and characters of representative isolate ZJ-1**. **(A)** Typical disease symptoms of ascochyta blight on pea leaves and pods in field. **(B)** Colony morphology of ZJ-1 on PDA plate after 9-day incubation. **(C)** The disease symptoms caused by the ZJ-1 inoculums on pea leaves and pods. **(D)** The phylogentic tree of ZJ-1, constructed by the neighbor-joining method with Molecular Evolutionary Genetics Analysis version 4.0, based on the ITS sequence (upper) and *G3PDH* gene (bottom).

The infected field pea plant tissues collected from six sites in Zhejiang Province presented typical ascochyta blight symptoms, including black necrotic spots on leaves and pods, blackening at the base of the stem, and foot rot in seedlings (Figure 1A). A total of 65 single-pycnidiospore isolates were obtained from infected tissue samples. All of these isolates displayed dense and felty colony morphologies on the PDA plates. Colony color tended to gray and darken with age from the center to the edge (Figure 1B). These colony morphological features resembled those reported for *Ascochyta* species. The virulence of all isolates was determined on pea leaves and pods. Typical symptoms are shown in Figure 1C; the inoculums caused brown lesions on leaves and pods with an additional wide yellowish margin on pods. There was no significant difference in the virulence among all tested strains based on the size of leaf lesions (data not shown). Our results indicated that all 65 isolates were pathogenic and associated with the disease.

To identify these isolates, partial regions of the ITS1-4 and *G3PDH* genes were amplified and sequenced. The ITS sequences obtained from the 65 isolates were identical to each other and were 507 bp in length excluding the two primers. Therefore, we randomly picked one isolate designated ZJ-1 for further study. Strain ZJ-1 was isolated from our breeding field in Haining. A blastn search showed that the 507 bp fragment of ZJ-1 was 100% identical to the ITS sequences of *A. pinodes* and *A. pinodella* deposited in GenBank. Phylogenetic analyses of the ITS sequences were conducted using the neighbor-joining method with Molecular Evolutionary Genetics Analysis version 4.0. Based on the ITS sequence of ZJ-1 and 6 other *Ascochyta* species from the NCBI GenBank (*A. pinodes*, FJ032644; *A. pinodella*, FJ032641; *A. pisi*, EU754131; *A. herbarum*, AF218792; *A. medicaginis*, AF079775; *A. exigua*, AY927784), we constructed a phylogenetic tree for ZJ-1 and 6 sequenced *Ascochyta* species. ITS analysis indicated that the closest species to ZJ-1 is either *A. pinodes* or *A. pinodella* (Figure 1D, upper). Then, we designed primers to amplify, and subsequently sequenced, part of the *G3PDH* gene. All isolates were shown to contain an identical 467 bp fragment of the *G3PDH* sequence. The partial sequence of *G3PDH* gene in ZJ-1 showed 100% identical to that from *A. pinodes* strain MP2 (DQ383976). ZJ-1 was grouped into the *A. pinodes* clade in the phylogenetic tree constructed with the *G3PDH* gene. Taken together, all isolates were genetically identical and classified as *A. pinodes* based on colony morphologies, disease symptoms, and ITS and *G3PDH* sequences. *A. pinodes* was the main pathogenic fungi to cause ascochyta blight in field peas in this area.

### Morphological and physiological characteristics of ZJ-1

**Figure 2:**
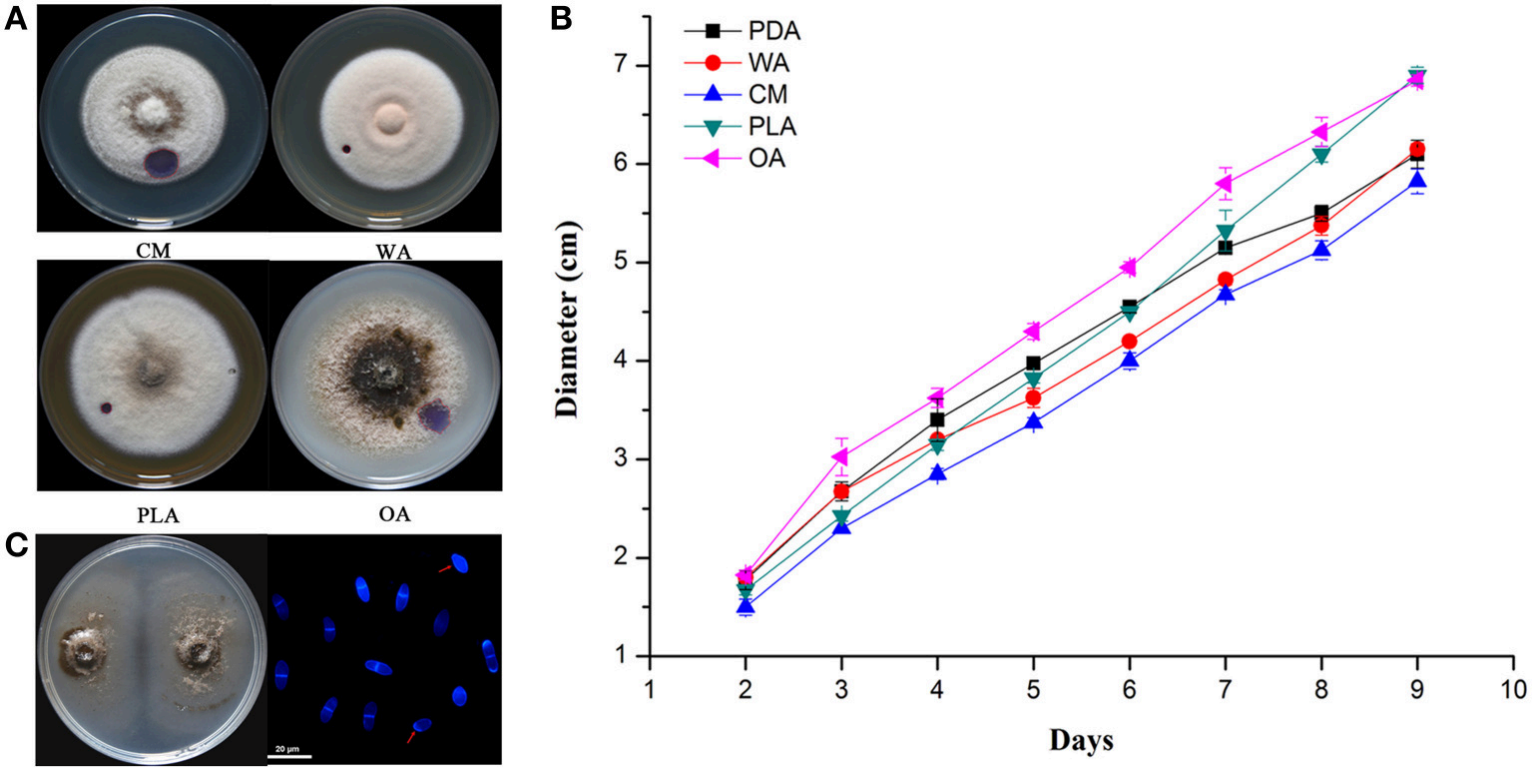
**Morphological and physiological characteristics of isolate ZJ-1. (A)** The colonies morphology of ZJ-1 on different media after 9-day incubation. **(B)** The growth rate of ZJ-1 on CM, WA, PLA and OA plates. The diameter of each colony was measured every 24 h. **(C)** Conidia formation on 1/3 PDA plates after 2 weeks incubation. The conidia were stained with calcofluor white and visualized by Leica TCS SP5 imaging system (Wetzlar, Hesse-Darmstadt, Germany). Bar was 20 μm.

The color of ZJ-1 colonies varied on different media. In general, colonies on PDA and OA were darker gray, most turning black at maturity, than those on CM, WA and PLA media (Figure 2A). The mycelia on WA and PLA media were much denser than those on other media, where the indicator, a droplet of 2.5% bromophenol blue water solution, caused smaller water-soaked areas (Figures 1B, 2A). Linear growth of ZJ-1 showed significantly different rate on various tested media. The results indicated that the mycelia growth rate of ZJ-1 on plant material media, including OA, PLA and PDA, was faster than that on CM and WA (Figure 2B). Among these media, ZJ-1 grew fastest on OA media and the colony expanded 7.61 ± 0.06 cm per day. ZJ-1 could produce conidia on 1/3 PDA plates after 2 weeks of incubation. The conidia were (13.1 ± 1.9) × (3.5 ± 1.5) μm in size and most harbored one septum, or occasionally no septum (Figure 2C).

**Figure 3:**
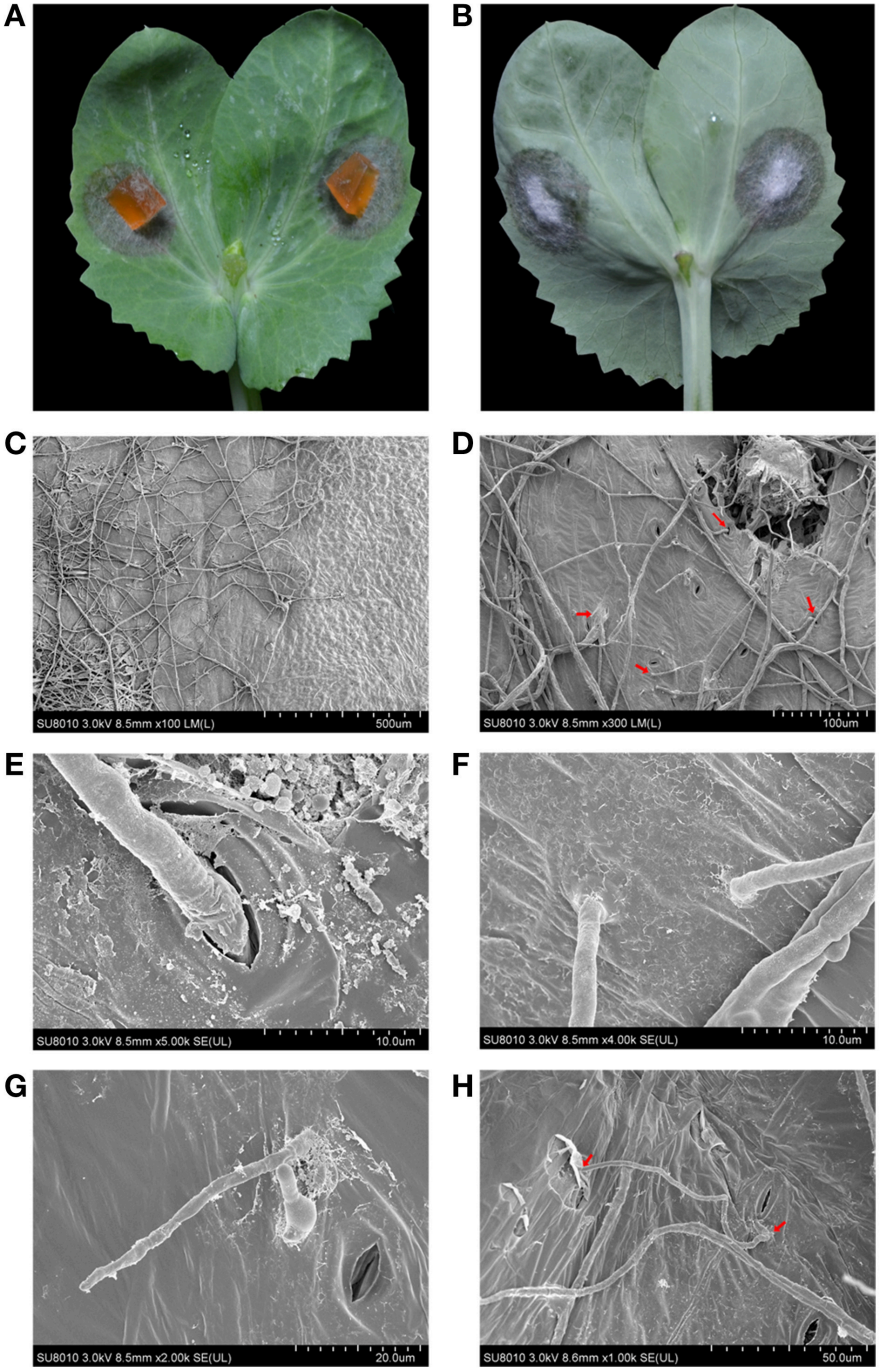
**The infection patterns of ***A. pinodes*** ZJ-1 were visualized by scanning electron microscope. (A)** The necrotic lesions on the surface of pea leaves (Zhewan-1 cultivar) caused by ZJ-1. **(B)** The mycelia of ZJ-1 penetrated the leaves and formed velvet on the backside. **(C)** The light-dark contrasts and physical patterns of boundary between healthy and necrotic tissue. **(D)** The mycelia patters and penetration structures of ZJ-1 formed on the leaves. **(E)** The mycelia penetrated the leaves across the stomas. **(F,G)** The mycelia of ZJ-1formed specific penetration structures and directly pierced leaves. **(H)** The infective hyphae were able to shuttle back and forth on the leaves and subsequently caused the brownish necrosis and chlorosis symptoms. The voltage and bars were indicated at the bottom of each panel.

The mycelial inoculums caused necrotic lesions on the surface of pea leaves, and the mycelia were able to penetrate the leaves and form velvet on the backside (Figures 3A,B). To gain an insight into the details of penetration, the infected leaves were fixed, dehydrated and observed using a scanning electron microscopy (SEM). The boundary between healthy and necrotic tissue was clear and showed significantly different light/dark contrasts and physical patterns (Figure 3C). As shown in Figures 3D,E, the mycelia of ZJ-1 expanded on the surface of the leaves and penetrated the leaves across the stomas (Figures 3D,E). Moreover, the mycelia formed specific penetration structures and directly pierced leaves (Figures 3F,G). The infective hyphae were able to shuttle back and forth on the leaves and subsequently cause the brownish necrosis and chlorosis symptoms (Figure 3H).

### Cultivar susceptibility

To screen the resistant cultivars for the ability to control ascochyta blight in peas, we evaluated the susceptibility of 23 pea cultivars that we obtained, including the cultivars Zhewan-1 and Xiangwan-1, which are widely grown in this area. All tested cultivars were infected with the ZJ-1 strain but displayed different resistance to ascochyta blight (Table 1). Leaf lesions were significantly different on the various cultivars, ranging from 1.70 to 4.08 cm in radium. Among them, Zhengzhu Lv had the smallest lesion size (average 1.70 cm), while D8341 showed the most susceptibility to ZJ-1. Zhewan-1 and Xiangwan-1 presented moderate resistant to ascochyta blight. We also randomly picked another 5 isolates from the remaining 64 isolates and evaluated the virulence of tested cultivars. There was no significant different in virulence compared with ZJ-1 (data not shown). Therefore, our results indicated that none of the tested cultivars were resistant to local ascochyta blight fungus.

### Sensitivity of ZJ-1 to fungicides

The effective concentrations (EC_50_) of 14 commonly used fungicides belonging to 8 categories for ZJ-1 were determined *in vitro*. ZJ-1 was sensitive to all tested fungicides with different EC_50_ values (Table 2). The EC_50_ values for carbendazim and thiophanate-methyl were 1.318 and 16.163 μg/mL, respectively, although both of them belong to the benzimidazole group. ZJ-1 was highly sensitive to sterol biosynthesis-inhibiting (SBI) fungicides, including tridemorph, difenoconazole, prochloraz, tebuconazole and propiconazole, with EC_50_ values that ranged from 0.005–1.15 μg/mL. Moreover, boscalid, penthiopyrad, fludioxonil, pyrimethanil, iprodione and prothioconazole also effectively inhibited mycelial growth of ZJ-1, with EC_50_ values ranging from 0.058 to 6.178 μg/mL. However, chlorothalonil was less efficient against ZJ-1 (EC_50_ = 13.969 μg/mL) isolated from Haining, where chlorothalonil has been extensively sprayed to control ascochyta blight in peas. These data provided documentation of the sensitivity of 14 fungicides against the representative strain ZJ-1, and most of them showed high activity toward this pathogen. The other 5 tested isolates showed identical susceptibility toward all tested fungicides. While we cannot properly refer to this as a study of baseline sensitivity for *Ascochyta pinodes*, it will provide a frame of reference for any future issues with fungicide sensitivity or resistance found in the region.

### Screening bacterial biocontrol agents against ZJ-1

**Figure 4:**
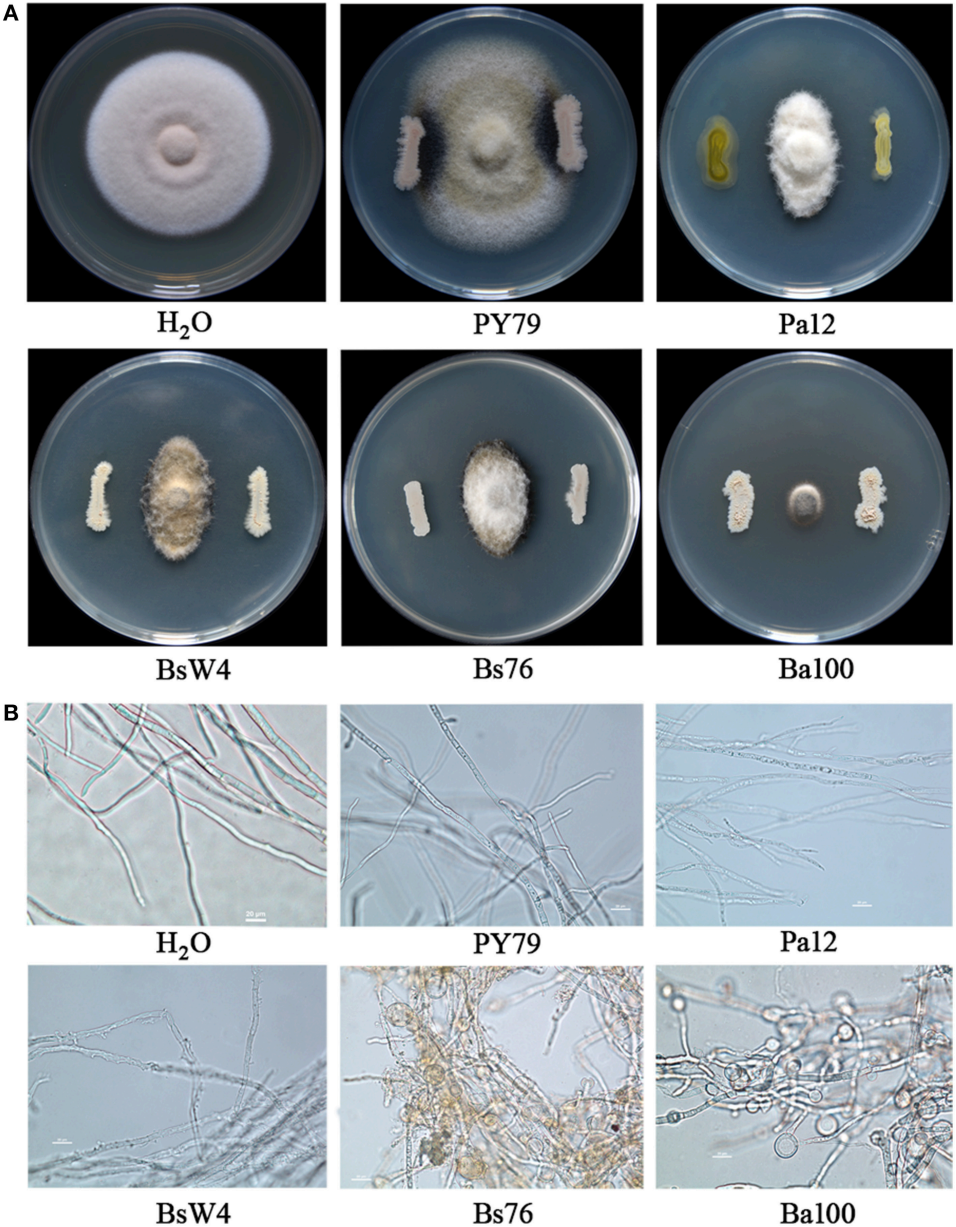
**The antagonistic activity of biocontrol bacterial strains and their cell free supernatants against ***A. pinodes*** ZJ-1. (A)** Antagonistic activity of Pa12, BsW4, Bs76 and Ba100 against ZJ-1 after co-culture on WA plates. **(B)** The mycelial features of ZJ-1 after biocontrol strain cell free supernatant treatment. The *Bacillus subtilis* PY79 and water was used as control.

Cultivable bacterial species were found to be very abundant in pea tissues and rhizosphere soil. After 2 days incubation, 155 and 261 isolates were picked from tissues and rhizosphere soil samples, respectively, according to colony morphology. The antagonistic activity of all isolates against ZJ-1 was examined on Waksman's Agar. In total, appropriately 10% (43) isolates showed various degree of suppression toward ZJ-1. Among these, four isolates showed very strong antagonistic activity, as evidenced by their formation of ZJ-1 inhibition zones > 10 mm (from the edge of bacterium to fungus) in the *in vitro* assay (Figure 4A). Biocontrol agents were identified with the 16S rRNA sequence. A 1402-bp PCR fragment was amplified with primers fD1/rP2 from them respectively, and subsequently 16S rRNA sequences were deposited in GenBank under accession numbers KU373080 to KU373083. The blastn results indicated that they were classified into two genuses, *Pantoea* sp. and *Bacillus* sp. We further constructed phylogenetic trees with 16S rRNA sequences for these four isolates and various whole genome sequenced reference strains from NCBI GenBank. The results indicated that the closest species to Ph12 was *Pantoea agglomerans* (Figure S2A), while Ba100 was cluster with the biocontrol agent *Bacillus amyloliquefaciens* FZB42. The 16S rRNA sequences of BsW4 and Bs76 presented 99% similarity to *Bacillus subtilis*, and closest with *B. subtilis* GB03 (Figure S2B). Taken together, biocontrol strains Ph12, Ba100, BsW4, and Bs76 were identified as *P. agglomerans, B. amyloliquefaciens*, and *B. subtilis*, respectively. As the results shown in Figure 4A, the bacterial biocontrol agents Pa12 (*P. agglomerans*), BsW4 (*B. subtilis*), Bs76 (*B. subtilis*), and Ba100 (*B. amyloliquefaciens*) significantly suppressed the mycelial growth of ZJ-1 compared with the control strain *B. subtilis* PY79. Ba100 presented the strongest inhibition against mycelial growth of fungus on WA plates after co-culture. The obvious inhibition zone between bacterial agents and fungus indicated that bacteria could produce diffuse active compounds to kill fungus. Therefore, we tested the bioactivity of cell free supernatants (CFW) toward ZJ-1. Treatment of hyphae with Pa12 CFW caused the uneven distribution of cellular contents and increased partial vesiculation of the membrane. The BsW4 CFW lysed the hyphae and caused the leakage of intracellular components. Hyphae treated with the Bs76 and Ba100 CFW displayed misshapen and severely distorted and condensed structures with increased vacuole sizes and conglobated apical tips (Figure 4B).

### Efficacy of foliar fungicides and biocontrol agents

Under greenhouse conditions, the bacterial suspension and fungicides were applied before ZJ-1 inoculation. After 2 weeks of incubation, ascochyta blight disease was severe, with disease severity of 78.22 and 83.11% in control treatments. However, the disease severity in all treatments was significantly lower than that in the untreated (Table 3). All bacterial biocontrol agents showed more than 65% biocontrol efficacy toward this disease. Among them, the biocontrol efficacy of Ba100 was the best at greater than 80% and was close to the efficacy of carbendazim under greenhouse conditions. Fludioxonil had the highest efficacy against this disease, which reached nearly 95%.

**Table 3:** **Ascochyta blight severity on pea plants and biological efficacy of bacterial biocontrol agents and fungicides application under greenhouse and in the field conditions**.

**Treatments**	**Greenhouse**				**Field**			
	**Repeat 1**		**Repeat 2**		**Repeat 1**		**Repeat 2**	
	**Severity (%)**	**Efficacy (%)**	**Severity (%)**	**Efficacy (%)**	**Severity (%)**	**Efficacy (%)**	**Severity (%)**	**Efficacy (%)**
Pa12	25.78±2.04^b^^※^	67.05±2.60^f^	24.44±2.78^b^	71.48±2.53^d^	10.89±2.04^b^	52.88±8.81^c^	9.56±1.39^b^	41.89±8.44^c^
BsW4	16.00±3.53^c^	79.55±4.51^e^	16.00±2.67^c^	80.75±3.21^c^	8.22±1.68^bc^	64.42±7.26^b^	7.56±0.77b^c^	54.05±4.68^b^
Bs76	14.22±1.54^cd^	81.82±1.97^de^	17.33±4.00^c^	80.75±2.78^c^	8.00±1.15^bc^	65.38±5.00^b^	7.11±1.39^c^	56.76±8.44^b^
Ba100	12.44±2.04^de^	84.09±2.60^cd^	13.78±2.04^c^	82.71±1.72^c^	6.89±1.68^cd^	70.19±7.26^b^	6.22±0.77^c^	62.16±4.68^b^
Carbendazim	10.22±0.77^ef^	86.93±0.98^bc^	8.44±2.04^d^	89.66±2.41^b^	4.22±0.38^de^	81.73±1.67^a^	2.22±0.38^d^	86.49±2.34^a^
Tebuconazole	8.00±2.67^f^	89.77±3.41^b^	6.22±0.77^d^	92.69±0.82^ab^	3.78±0.77^de^	83.65±3.33^a^	1.56±0.38^d^	90.54±2.34^a^
Boscalid	9.78±2.04^ef^	87.50±2.60^bc^	8.44±1.54^d^	89.13±0.62^b^	4.00±0.67^de^	82.69±2.88^a^	1.78±0.38^d^	89.19±2.34^a^
Fludioxonil	4.44±0.77^g^	94.32±0.98^a^	4.00±1.33^d^	94.65±0.93^a^	2.44±0.38^e^	89.42±1.67^a^	1.11±0.38^d^	93.24±2.34^a^
Iprodione	8.44±0.77^f^	89.20±0.98^b^	7.56±0.77^d^	90.55±0.31^b^	4.22±0.38^de^	81.73±1.67^a^	2.22±0.77^d^	86.49±4.68^a^
Untreated	78.22±2.04^a^		83.11±3.85^a^		23.11±5.00^a^		16.44±2.69^a^	

※ *The data were analyzed using Fisher's protected least significant difference test (P = 0.05) in SAS (SAS version 8.0; SAS Institute, Cary, NC, USA). The same letters are not significantly different (P = 0.05)*.

The field experiments were conducted in 2015, and ascochyta blight occurred in pea crops at both experimental sites. In general, the biological efficacies of all fungicide treatments were consistent with those observed under greenhouse conditions. However, the biocontrol efficacy of bacterial agents was lower in the field, compared to that seen under greenhouse conditions (Table 3). These results indicated that all five tested fungicides could be effectively used to treat and control ascochyta blight infield peas, and four selected bacterial agents also significantly reduced the disease severity and could be applied as an alternative approach to disease control in this area.

## Discussion

The causal agents of ascochyta blight in field peas are diverse, including *Ascochyta pinodes, Phoma pinodella, Asco chyta pisi, Phoma koolunga, Phoma herbarum*, and *Phoma glomerata*. These pathogens can occur together within one pea field and even on one single plant (Hare and Walker, 1944). However, the ascochyta fungal population structures and distribution are varied in different regions, which may be due to geography, host selection pressure and environmental conditions. *A. pinodes* was the main pathogen infecting peas in Canada and France (Moussart et al., 1998; Bretag et al., 2006; Tivoli and Banniza, 2007; Gossen et al., 2011; Le May et al., 2012; Ahmed et al., 2015). *A. pinodes* and *P. pinodella* were widespread in all tested regions in Australia, while *P. koolunga* was commonly detected in soil from South Australia (Bretag, 1991; Davidson et al., 2009, 2011; Li et al., 2011; Tran et al., 2014). However, in 2010 in a field pea blackspot disease screening nursery at Medina, Western Australia, approximately 25% of isolates were *P. herbarum* and 1% of isolates were *P. glomerata* (Tran et al., 2014). In Lithuanian, *A. pisi* was the prevalent specie and accounted for nearly half of the pathogens isolated from Ascochyta complexes, whereas at some sites, the prevalent species were *A. pinodes* and *P. pinodella* (Cesnuleviciene et al., 2014). Information on the composition of the pathogen species causing ascochyta blight in field peas has not been determined in Zhejiang province, China. In this study, we collected 65 isolates from 5 sites in this area and identified them based on ITS and *G3PDH* sequence alignments. Previous studies (Fatehi et al., 2003; Peever et al., 2007; Davidson et al., 2009; Tadja et al., 2009) have indicated that the ITS sequences can be an effective tool to separate *A. pinodes* and *P. pinodella* from *A. pisi* and *P. koolunga*, whereas they did not allow differentiation between *A. pinodes* and *P. pinodella*. Consistent with this finding, the ITS sequences of all 65 isolates showed 100% identity to both *A. pinodes* and *P. pinodella* (Figure 1C). Conidia differentiation is commonly used to separate these two fungi, although identification on the basis of microscopic examination can be difficult because of isolate-to-isolate variation (Bretag et al., 2006). Random amplification of polymorphic DNA (RAPD) analysis (Onfroy et al., 1999) and restriction fragment length polymorphism (RFLP) analysis of mitochondrial DNA were reported to be useful tools for distinguishing *A. pinodes* from *P. pinodella* (Fatehi et al., 2003). However, band patterns similar to those reported were not easily repeatable (Liu et al., 2013). It has been reported that it is possible to discriminate *A. pinodes* and *P. pinodella* through phylogenetic analysis of *G3PDH* sequences (Peever et al., 2007). Therefore, we sequenced the partial of *G3PDH* gene from the isolates and constructed a phylogenetic tree, and subsequent results indicated that all isolates were *A. pinodes* (Figure 1D). The identification based on molecular techniques was confirmed using morphology features of the representative isolate, ZJ-1(Figure 2C). In addition, *P. pinodella* is a plant quarantine pathogen in China. Taken together, *A. pinodes* was the prevalent and majority specie causing ascochyta blight in the field peas in our tested areas.

Here, we observed first the infection structure of *A. pinodes* on the pea leaves and found that the fungus formed penetration structures like other fungal plant pathogens, such as *Fusarium graminearum, Magnaporthe oryzae*, and *Mycosphaerella graminicola*. Generally, gene families involved in cell wall degradation are responsible for the biotrophic phase of penetration in fungal plant pathogens (Yun et al., 2000; Martin et al., 2010). Therefore, the extracellular endoglucanase and secreted cellulose enzyme activities of ZJ-1 were analyzed on agar plates supplemented with beta-1, 3 glucans orsodium carboxymethyl cellulose as the sole carbon source. There were no or very weak degradation halorings around the mycelia of colonies after 1 week of incubation. Moreover, the strain grew slowly (data not shown). These observations indicated that, in *A. pinodes* ZJ-1, the activities of cell wall-degrading enzymes were very low, and an alternative mechanism may be involved in the process of degrading pea tissues. This hypothesis was indirectly supported by genome information from *Mycosphaerella graminicola* (Goodwin et al., 2011), a fungal wheat pathogen, which is the same specie as *A. pinodes* (teleomorph: *Mycosphaerella pinodes*). An interesting feature of the *M. graminicola* genome compared to other sequenced plant pathogens is that it contains very few genes for enzymes that break down plant cell walls, but it has expanded peptidases and alpha amylases, which are involved in the degradation of proteins. This finding suggests that the stealth pathogenesis of *M. graminicola* probably involves degradation of proteins rather than carbohydrates to evade host defenses during the biotrophic stage of infection and may have evolved from endophytic ancestors (Kema et al., 1996; Goodwin et al., 2011). *A. pinodes* may utilize a similar pathogenesis process in peas. Genome sequencing of *A. pinodes* is underway to elucidate this mystery.

The search for resistant cultivars and resources against ascochyta blight in peas has been ongoing since the 1940s in Georgia, USA (Stuckey, 1940). In that study, a total of 208 lines were screened in the field and found that the Austrian Winter Pea line was the most resistant. However, Weimer (1947) screened a large pea collection over several years and found no resistance in cultivated peas, including Austrian Winter Peas. Matthews et al. (1985) screened the pea collection of the John Innes Institute, U. K., and he found that none were resistant against *A. pinodes*. Bretag (1989) tested 32 pea lines in the field over 2 years and found that only one variety, Rondo, showed a significant level of resistance. Kraft et al. (1998) screened approximately 2936 germplasms from the USDA collection from 1991 to 1995 in Ireland and New Zealand. Their results suggested that five lines, PI 142441 (Peru), PI 142442 (Peru), PI 381132 (Ethiopia), PI 404221 (Russia), and PI 413691 (Hungary), were resistant in both Ireland and New Zealand. Only one line, PI 413691, showed consistent partial resistance in a Canadian study (Xue and Warkentin, 2001), where 335 accessions representing 30 countries were tested, and 51 lines showed partial resistance. Francis et al. (2000) reported that approximately 40 lines out of 500 lines displayed partial resistance to ascochyta blight using field screening in Ethiopia in 1998. Fondevilla et al. (2005) found little resistance in cultivated pea types but useful resistance in the wild peas *P. sativum* ssp. *elatius* and *P. sativum* ssp. *syriacum* in a study involving 78 accessions. Zhang et al. (2006) found that most lines showed a low level of partial resistance in a study involving 558 lines. To date, 30 named pea varieties have been reported to have some resistance to *A. pinodes* (Khan et al., 2013). In this present study, we tested the level of resistance of 23 lines against *A. pinodes* ZJ-1 using the leaf assay. Consistent with the above-mentioned studies, our result indicated that all tested lines were susceptible to ZJ-1, although the disease severities were significantly different. All studies conducted so far have suggested that both cultivated and wild pea genotypes as well as sub-species of *P. sativum* do not show high levels of robust resistance against ascochyta blight. Incorporating traditional breeding programs and biotechnologies will accelerate the progress of breeding and selecting ascochyta blight-resistant cultivars.

Successful colonization and antifungal production on the plant surface or within plant tissues are critical for biocontrol agents to control disease. In this study, the results from *in vitro* and *in vivo* evaluations demonstrated that four biocontrol agents isolated from pea fields were able to control ascochyta blight caused by *A. pinodes* ZJ-1. *Bacillus subtilis* and *Bacillus amyloliquefaciens* are well known for their biocontrol of fungal and bacterial diseases. The main mechanism is the production of a great abundance of antibiotics with an amazing variety of structures and activities by *Bacillus* sp. (Stein, 2005). Among these antimicrobial compounds, cyclic lipopeptides (LPs) of the iturin, fengycin (or plipastatin) and bacillomycin families display strong *in vitro* antifungal activities against a wide variety of fungi. The supernatant of *B. subtilis* strains BsW4 and Bs76 and *B. amyloliquefaciens* Ba100 showed high inhibition of ZJ-1 mycelial growth and caused a misshapen morphology. Hyphae treated with supernatants from Bs76 and Ba100 displayed condensed structures with increased vacuole sizes and conglobated apical tips, which were similar to the morphologies of *F. graminearum* mycelia treated with iturin and plipastatin (fengycin) (Gong et al., 2015). The TOF-MS data indicated that LPs of the iturin C, fengycin A and bacillomycin D families, produced by these three *Bacillus* sp. strains, were the main antifungal compounds against ZJ-1(Table S1, Supplemented Data). Colonization and persistence are considered major challenges for the implementation of bacterial biocontrol agents. Bacterial biofilms are multicellular communities in which cells are held together by an extracellular matrix that is composed mainly of exopolysaccharides, proteins and nucleic acids (Branda et al., 2001). It has been reported that *Bacillus* sp. biofilms are critical for colonization and biocontrol efficacy for plant diseases (Bais et al., 2004; Chen et al., 2012a,b). Here, all three *Bacillus* sp. strains can form robust biofilms in biofilm-inducing medium (Figure S3), that was ideal for bacterial colonization on the surfaces of plant tissues. In addition, this was the first report that the *P. agglomerans* strain has the potential for development as a biofungicide for management of ascochyta blight in field peas. Two antibiotics, pantocin A and pantocin B, have been identified from biocontrol strain *P. agglomerans* Eh318 and have demonstrated antibacterial activities (Sutton and Clardy, 2001; Jin et al., 2003). However, antifungal compounds from *P. agglomerans* have not been well studied. It will be interesting in the future to identify the antifungal composites produced by Pa12.

In conclusion, our results indicated that *A. pinodes* was the prevalent specie causing ascochyta blight in the field peas in Zhejiang Province. All pea cultivars grown in tested areas were susceptible to the fungus. Fortunately, most of the tested fungicides (11 out of 14) showed high activity toward the pathogen with EC_50_values < 5 μg/mL. Moreover, fungicides, including tebuconazole, boscalid, iprodione, carbendazim, and fludioxonil, displayed greater than 80% disease control efficacy under field conditions. Bacterial biocontrol agents isolated in this study also have the potential for ascochyta blight disease management. An approach using chemical fungicides in conjunction with biocontrol agents is being developed to synergistically suppress this disease.

## Author contributions

Conceived and designed the experiments: NL, YG. Performed the experiments: NL, SX, GZ, QH, ZF. Analyzed the data: NL, XY, WM. Wrote the paper: NL, YG.

### Conflict of interest statement

The authors declare that the research was conducted in the absence of any commercial or financial relationships that could be construed as a potential conflict of interest.

## Footnotes

^1^Available online at: http://faostat.fao.org

## Supplementary material

Supplementary Figure 1 **Production of dry peas (A) and green peas (B) in the 10 most productive countries**. The data were obtained from FAO, 2015.

Supplementary Figure 2 **Identification of four biocontrol strain Ph12, Ba100, BsW4 and Bs76**. The phylogenetic trees were generated by the neighbor-joining method with the MEGA 4.1 software using the 16S rRNA sequences. **(A)** The phylogenetic tree of Ph12 and following *Pantoea* spp.: *Pantoea gaviniae* LMG253 (AB907786), *Pantoea eucrina* ATCC27998 (FJ611863), *Pantoea ananatis* ATCC27995 (FJ611812), *Pantoea septica* LMG5345 (NR_116244), and *Pantoea agglomerans* Eh318 (FJ611804). **(B)** The phylogenetic tree of Ba100, BsW4 and Bs76, and following *Bacillus* spp.: *Bacillus amyloliquefaciens* FZB42 (NR_075005), *Bacillus subtilis* GB03 (DQ683077), *Bacillus pumilus* SAFR-032 (gi|987861389), *Bacillus infantis* NRRL B-14911 (gi|549473062), *Bacillus cereus* ATCC 14579 (NR_074540), *Bacillus anthracis* Cvac02 (gb|CP008853), *Bacillus thuringiensis* XL6 (gb|CP013000), *Bacillus mycoides* ATCC 6462 (gi|754380545), and *Bacillus licheniformis* 12759 (gi|737357300).

Supplementary Figure 3 **Biofilm formation features of *Bacillus* sp. strain BsW4, Bs76 and Ba100 in biofilm inducible medium LBGM**. The pellicle biofilm formation by strain BsW4, Bs76 and Ba100 cells inoculated in LBGM medium after 3 days of incubation at 30°C (upper panels). The details of pellicles were also examined by using a stereomicroscope (lower panels).

Supplementary Table 1 **Identified lipopeptides produced from *Bacillus* sp. strains by TOF-MS**.
