# Supplementary figures and images for: Studies on the Control of Ascochyta Blight in Field Peas (Pisum sativum L.) Caused by Ascochyta pinodes in Zhejiang Province, China
Source: Front Microbiol. 2016 Apr 12;7:481. doi: 10.3389/fmicb.2016.00481 (PMC4828446; doi:10.3389/fmicb.2016.00481)

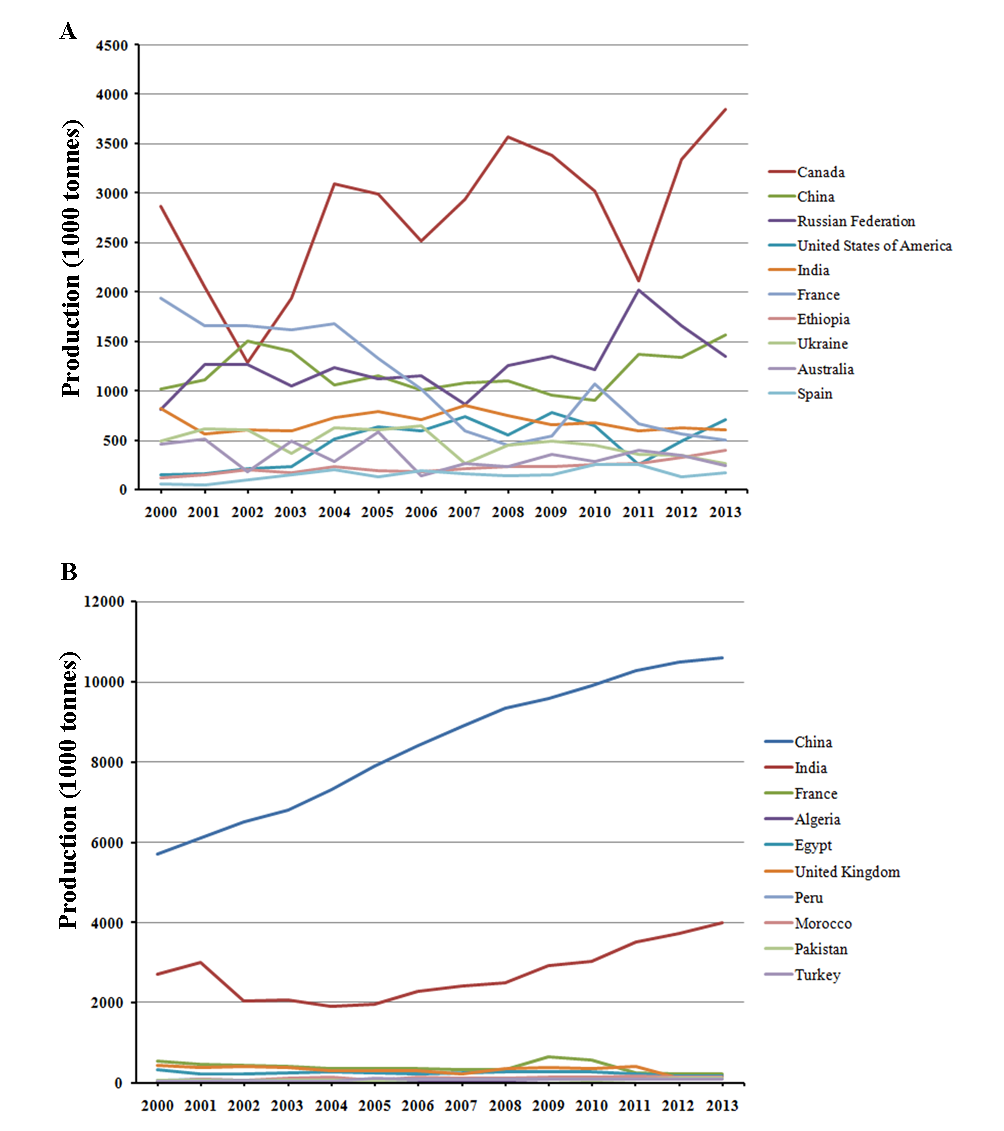

Supplement: Supplementary Figure 1 — Production of dry peas (A) and green peas (B) in the 10 most productive countries. The data were obtained from FAO, 2015. [file Image1.TIF]

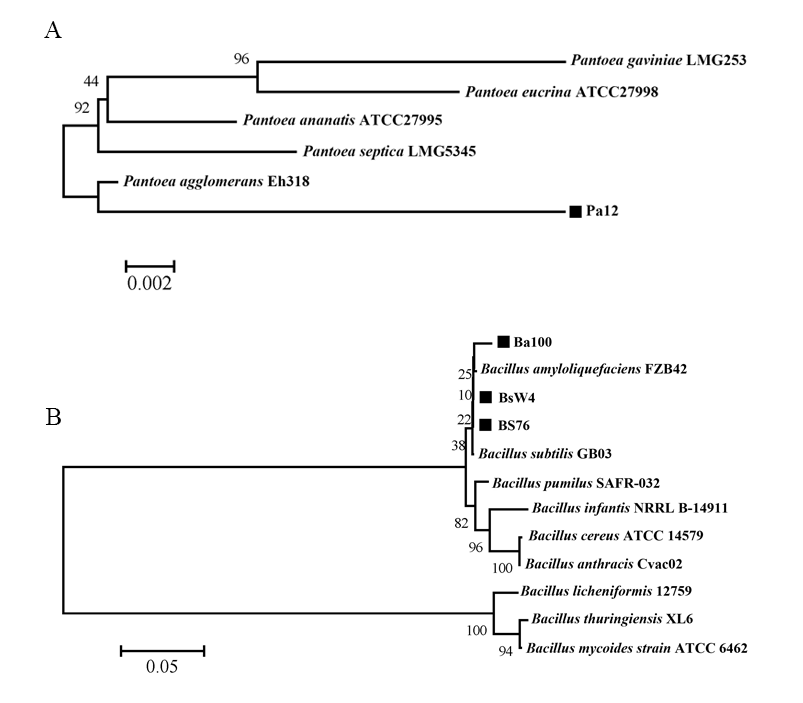

Supplement: Supplementary Figure 2 — Identification of four biocontrol strain Ph12, Ba100, BsW4 and Bs76. The phylogenetic trees were generated by the neighbor-joining method with the MEGA 4.1 software using the 16S rRNA sequences. (A) The phylogenetic tree of Ph12 and following Pantoea spp.: Pantoea gaviniae LMG253 (AB907786), Pantoea eucrina ATCC27998 (FJ611863), Pantoea ananatis ATCC27995 (FJ611812), Pantoea septica LMG5345 (NR_116244), and Pantoea agglomerans Eh318 (FJ611804). (B) The phylogenetic tree of Ba100, BsW4 and Bs76, and following Bacillus spp.: Bacillus amyloliquefaciens FZB42 (NR_075005), Bacillus subtilis GB03 (DQ683077), Bacillus pumilus SAFR-032 (gi|987861389), Bacillus infantis NRRL B-14911 (gi|549473062), Bacillus cereus ATCC 14579 (NR_074540), Bacillus anthracis Cvac02 (gb|CP008853), Bacillus thuringiensis XL6 (gb|CP013000), Bacillus mycoides ATCC 6462 (gi|754380545), and Bacillus licheniformis 12759 (gi|737357300). [file Image2.TIF]

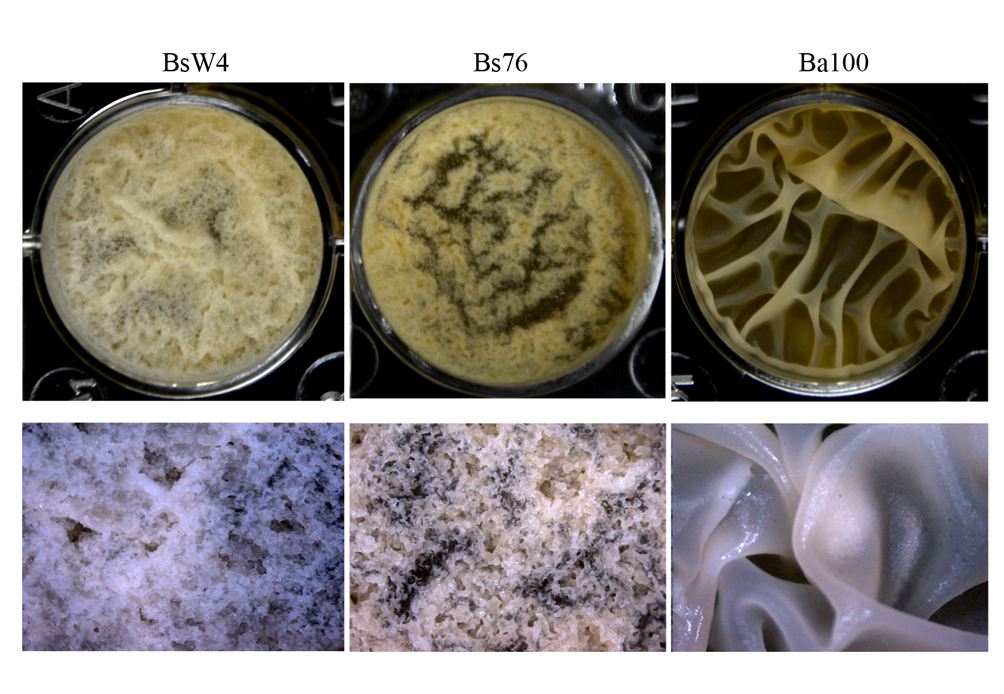

Supplement: Supplementary Figure 3 — Biofilm formation features of Bacillus sp. strain BsW4, Bs76 and Ba100 in biofilm inducible medium LBGM. The pellicle biofilm formation by strain BsW4, Bs76 and Ba100 cells inoculated in LBGM medium after 3 days of incubation at 30°C (upper panels). The details of pellicles were also examined by using a stereomicroscope (lower panels). [file Image3.TIF]
