# Supplementary material for: Studies on the Control of Ascochyta Blight in Field Peas (Pisum sativum L.) Caused by Ascochyta pinodes in Zhejiang Province, China
Source: Front Microbiol. 2016 Apr 12;7:481. doi: 10.3389/fmicb.2016.00481 (PMC4828446; doi:10.3389/fmicb.2016.00481)
Supplement: Supplementary Table 1 — Identified lipopeptides produced from Bacillus sp. strains by TOF-MS. [file Table1.DOCX]

**Table S1 Identified lipopeptides produced from *Bacillus* sp. strains by TOF-MS**

| **Sample** | **m/z** | **Ionic type** | **Compound** | **Molecular Formula** | **Molecular Weight** |
| --- | --- | --- | --- | --- | --- |
| Ba100 | 1030.644 | [M+Na]^+^ | Surfactin(C_11_) | C52H91N7013 | 1021 |
|  | 1044.6596 | [M+Na]^+^ | Surfactin(C_12_) | C53H93N7013 | 1035 |
|  | 1058.6755 | [M+Na]^+^ | Surfactin(C_13_) | C54H95N7013 | 1049 |
|  | 1080.553 | [M+Na]^+^ | Itutin C(C_12_) | C49H75N11015 | 1057 |
|  | 1088.6264 | [M+K]^+^ | Surfactin(C14) | C55H97N7013 | 1063 |
|  | 1094.5703 | [M+Na]^+^ | Itutin C(C_13_) | C50H77N11015 | 1071 |
|  | 1449.7939 | [M+H]^+^ | Fengycin A(C_15_) | C71H108N12020 | 1448 |
|  | 1463.8119 | [M+H]^+^ | Fengycin A(C_16_) | C72H110N12020 | 1462 |
|  | 1477.8257 | [M+H]^+^ | Fengycin A(C_17_) | C73H112N12020 | 1476 |
|  | 1491.8404 | [M+H]^+^ | Fengycin A(C_18_) | C74H114N12020 | 1490 |
|  | 1505.8405 | [M+H]^+^ | Fengycin A(C_19_) | C75H116N12020 | 1504 |
|  | 1513.8218 | [M+Na]^+^ | Fengycin A(C_18_) | C74H114N12020 | 1518 |
| Bs76 | 1030.644 | [M+Na]^+^ | Surfactin(C_11_) | C52H91N7013 | 1021 |
|  | 1044.6593 | [M+Na]^+^ | Surfactin(C_12_) | C53H93N7013 | 1035 |
|  | 1046.6663 | [M+K]^+^ | Surfactin(C_11_) | C52H91N7013 | 1021 |
|  | 1058.6754 | [M+Na]^+^ | Surfactin(C_13_) | C54H95N7013 | 1049 |
|  | 1066.54 | [M+Na]^+^ | Itutin C(C_11_) | C48H73N11015 | 1043 |
|  | 1080.556 | [M+Na]^+^ | Itutin C(C_12_) | C49H75N11015 | 1057 |
|  | 1081.5549 | [M+Na]^+^ | Bacillomycin D (C_13_) | - | 1044 |
|  | 1095.5676 | [M+Na]^+^ | Bacillomycin D (C_14_) | - | 1058 |
|  | 1463.8084 | [M+H]^+^ | Fengycin A(C_16_) | C72H110N12020 | 1462 |
|  | 1477.8272 | [M+H]^+^ | Fengycin A(C_17_) | C73H112N12020 | 1476 |
|  | 1491.84 | [M+H]^+^ | Fengycin A(C_18_) | C74H114N12020 | 1490 |
| BsW4 | 1030.6419 | [M+Na]^+^ | Surfactin(C_11_) | C52H91N7013 | 1021 |
|  | 1044.6585 | [M+Na]^+^ | Surfactin(C_12_) | C53H93N7013 | 1035 |
|  | 1046.6637 | [M+K]^+^ | Surfactin(C_11_) | C52H91N7013 | 1021 |
|  | 1058.6742 | [M+Na]^+^ | Surfactin(C_13_) | C54H95N7013 | 1049 |
|  | 1060.6791 | [M+K]^+^ | Surfactin(C_12_) | C53H93N7013 | 1035 |
|  | 1080.6563 | [M+Na]^+^ | Itutin C(C_12_) | C49H75N11015 | 1057 |
|  | 1088.6233 | [M+K]^+^ | Surfactin(C_14_) | C55H97N7013 | 1063 |
|  | 1102.6376 | [M+K]^+^ | Surfactin(C_15_) | C56H99N7013 | 1077 |
|  | 1463.8065 | [M+H]^+^ | Fengycin A(C16) | C72H110N12020 | 1462 |
|  | 1463.8389 | [M+H]^+^ | Fengycin A(C16) | C72H110N12020 | 1462 |
|  | 1491.8387 | [M+H]^+^ | Fengycin A(C18) | C74H114N12020 | 1490 |
