# Supplementary material for: Studies on the Control of Ascochyta Blight in Field Peas (Pisum sativum L.) Caused by Ascochyta pinodes in Zhejiang Province, China
Source: Front Microbiol. 2016 Apr 12;7:481. doi: 10.3389/fmicb.2016.00481 (PMC4828446; doi:10.3389/fmicb.2016.00481)
Supplement: Supplementary file 5 [file DataSheet1.DOCX]

**+TOF MS spectrum information of lipopeptides produced by *Bacillus* sp. strains**

**Spectrum from Ba100, +TOF MS (800-2000)**

**Spectrum from Bs76, +TOF MS (800-2000)**

**Spectrum from BsW4, +TOF MS (800-2000)**
